# Suppression of neurotransmission on gonadotropin-releasing hormone neurons in letrozole-induced polycystic ovary syndrome: A mouse model

**DOI:** 10.3389/fendo.2022.1059255

**Published:** 2023-01-09

**Authors:** Pravin Bhattarai, Santosh Rijal, Janardhan P. Bhattarai, Dong Hyu Cho, Seong Kyu Han

**Affiliations:** ^1^ Department of Oral Physiology, School of Dentistry and Institute of Oral Bioscience, Jeonbuk National University, Jeonju, South Korea; ^2^ Department of Neuroscience, University of Pennsylvania Perelman School of Medicine, Philadelphia, PA, United States; ^3^ Department of Obstetrics and Gynecology, Jeonbuk National University Medical School, Research Institute of Clinical Medicine of Jeonbuk National University-Biomedical Research Institute of Jeonbuk National University Hospital, Jeonju, South Korea

**Keywords:** γ-amino butyric acid, hypothalamic-pituitary-gonadal axis, kisspeptin, letrozole, patch-clamp, polycystic ovarian syndrome

## Abstract

**Objective:**

Polycystic ovarian syndrome (PCOS) is a heterogeneous endocrine disorder in reproductive-age women, characterized by the accretion of small cystic follicles in the ovary associated with chronic anovulation and overproduction of androgens. Ovarian function in all mammals is controlled by gonadotropin-releasing hormone (GnRH) neurons, which are the central regulator of the hypothalamic-pituitary-gonadal (HPG) axis. However, the impact on the neurotransmitter system regulating GnRH neuronal function in the letrozole-induced PCOS mouse model remains unclear.

**Methods:**

In this study, we compared the response of various neurotransmitters and neurosteroids regulating GnRH neuronal activities between letrozole-induced PCOS and normal mice *via* electrophysiological techniques.

**Results:**

Response to neurotransmitter systems like GABAergic, glutamatergic and kisspeptinergic were suppressed in letrozole-fed compared to normal mice. In addition, neurosteroids tetrahydrodeoxycorticosterone (THDOC) and 4,5,6,7-tetrahydroisoxazolo[5,4-c] pyridine-3-ol (THIP) mediated response on GnRH neurons were significantly smaller on letrozole-fed mice compared to normal mice. Furthermore, we also found that letrozole-fed mice showed irregularity in the estrous cycle, increased body weight, and anovulation in female mice.

**Conclusion:**

These findings suggest that PCOS is an endocrine disorder that may directly affect the neurotransmitter system regulating GnRH neuronal activity at the hypothalamic level and impact reproductive physiology.

## Introduction

Polycystic ovarian syndrome (PCOS) is a heterogeneous endocrine disorder in reproductive-age women which is characterized by the accretion of small cystic follicles in the ovary, increased GnRH pulsatility, hypersecretion of luteinizing hormone (LH), anovulation, hyperandrogenemia, and insulin resistance (1–3). However, the etiology of PCOS is poorly understood. Studies suggest that the onset of PCOS usually starts in the early stage of reproductive development due to excessive androgen production resulting in hormonal imbalance (4). PCOS has been emerging as one of the most common endocrine abnormalities in 5-10% of women causing severe reproductive problems such as the increased risk of infertility, adverse pregnancy outcomes, and metabolic syndrome associated with inflammatory risks and cardiovascular disease (3, 5). In addition, abdominal obesity, insulin resistance, and type 2 diabetes mellitus (DM2) are common in women with polycystic ovary syndrome (6).

Previous literature have shown a development of diverse spectrum of PCOS animal models to understand and investigate the etiology and pathology of PCOS (7). Reproductive and neuroendocrine traits possessed by rodent models of PCOS highly resemble the study on human PCOS (3). Several rodent models of PCOS can be imitated experimentally by administering various drugs and synthetic hormones, such as testosterone (T), dihydrotestosterone (DHT), dehydroepiandrosterone (DHEA), estrogens, and aromatase inhibitors (8–11). Letrozole, a non-steroidal aromatase inhibitor that blocks the conversion of androgens to estrogens resulting in increased endogenous testosterone level in the ovary (8), adrenal gland, and other peripheral tissues (12), and has proven to be a successful tool for making PCOS models in rodents (13–15). An administration of letrozole for 21 successive days induces many reproductive hallmarks of PCOS including large follicular cysts in the ovary, irregular estrous cycle, anovulation (8, 16), and increased body weight and fat (13).

The central regulator of the hypothalamic-pituitary-gonadal (HPG) axis, the gonadotropin-releasing hormone (GnRH) neurons, extend their axons to the median eminence, where they release GnRH in a pulsatile pattern (17, 18). In response to GnRH, the anterior portion of the pituitary produces pituitary gonadotropins: LH and follicle-stimulating hormone (FSH), which act on target organs to produce sexual gametes and steroidal hormones (19). Blood-borne LH and FSH are essential for normal gametogenesis and fertility in both males and females (20). Furthermore, the GnRH neuron-governed HPG axis directly regulates essential ovarian functions like steroidogenesis and folliculogenesis (21).

The GnRH neuronal network is crucial for pulsatile and surge modes of gonadotropin release (18). Multiple intrinsic and extrinsic factors, including neurosteroids, neurotransmitters, and neuropeptides, have a significant impact on GnRH pulsatility (22, 23). PCOS is linked with HPG axis dysfunction, possibly due to increased frequency and amplitude of the hypothalamic GnRH pulse generator (24) and gonadotropin release (25). However, to date, studies on PCOS and GnRH neuronal physiology are rare and typically use an androgenized PCOS model (26–28). Additionally, growing evidence supports the involvement of GnRH-regulatory neurotransmitters and neuropeptides in the pathogenesis of PCOS (28–30); however, lacks electrophysiological analysis for the letrozole-induced PCOS model. Therefore, we used a patch clamp electrophysiology approach to examine the response of GnRH-regulatory neurotransmitters like GABA, glutamate, kisspeptin, and neurosteroids on GnRH neurons of letrozole-induced PCOS mice.

## Materials and methods

### Animals

All animal handling procedures followed the guiding principles approved by Institutional Animal Care and Use Committee of Jeonbuk National University, CBNU 2016-64, and CBNU 2020-0122. GnRH-green fluorescent protein-tagged (GnRH-GFP; Strain: C57BL/6) mice (31), and ICR mice were housed under stable room temperature (23-26 °C) and an automatic 12-h light/12-h dark photocycle (lights on at 07:00 h) with free access to food and water.

### PCOS mouse model

**Figure 1:**
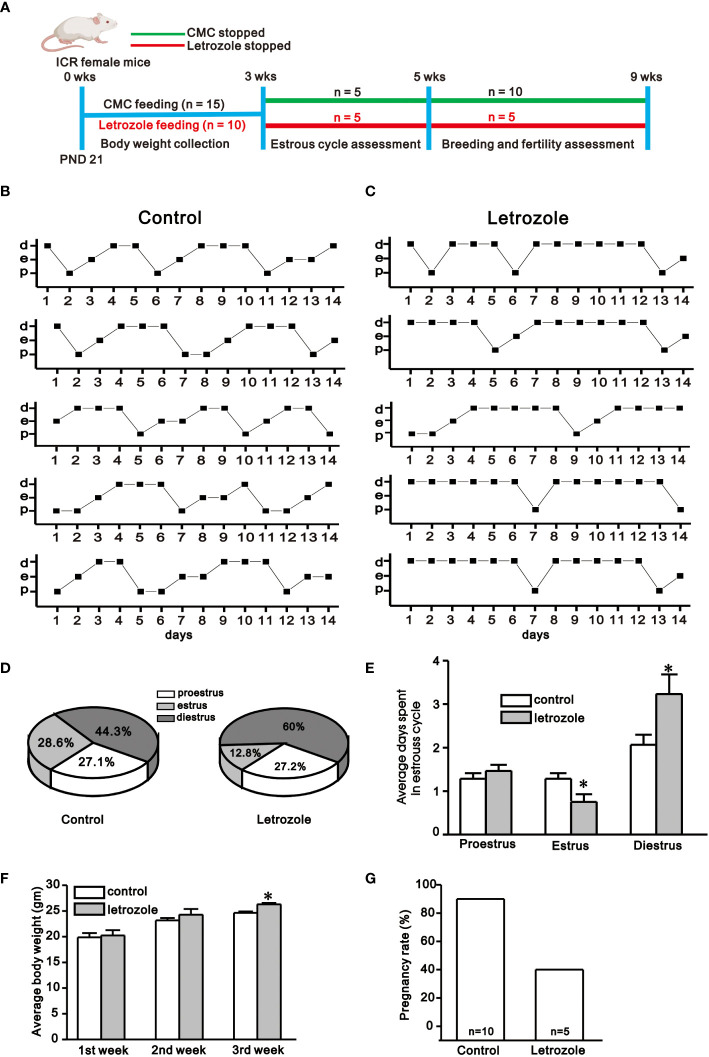
Assessment of estrous cyclicity, body weight, and pregnancy rate between control and letrozole-fed ICR mice. **(A)**. Experimental time line showing experimental design for the assessment of body weight, estrous cyclicity, and pregnancy rate between control and letrozole-fed ICR mice. **(B)** and **(C)**. Representative estrous cycles chart observed for 14 days between control and letrozole-fed mice (n = 5/group). (d, e, and p represent diestrus, estrus, and proestrus, respectively). **(D)**. Pie-chart depicts the percentage of estrous phases seen in the control and letrozole-fed mice. **(E)** A histogram comparing the average days spent in different stages of the estrous cycle during two weeks period between control and letrozole-fed mice. **(F)**. A bar graph comparing body weight between the control and letrozole-fed mice within three weeks. **(G)** A bar-diagram comparing the pregnancy rates between the control and letrozole stop groups. (*p < 0.05, unpaired *t*-test).

**Figure 2:**
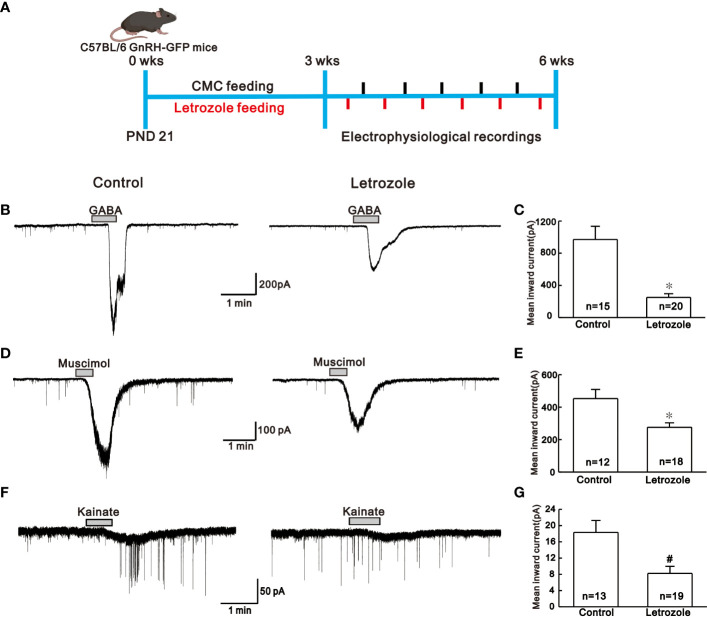
Comparison of the neurotransmitter response in the control vs letrozole-fed mice. **(A)**. Experimental time line showing experimental design for the electrophysiological recordings between control **(N = 5; n = 5 per batch)** and letrozole-fed **(N = 5; n = 6 per batch)** C57BL/6 GnRH GFP-tagged mice **(N and n represents number of batches and mice respectively) B, D,** and **F**. Representative traces for GABA, muscimol, and kainate-induced responses on GnRH neurons from control and letrozole-fed mice, respectively. **(C, E,** and **G)**. Histogram comparing the mean inward current induced by GABA, muscimol, and kainate on GnRH neurons from control and letrozole-fed mice, respectively. (*p < 0.05, unpaired *t*-test; ^#^ *p* < 0.05, Mann-Whitney test).

PCOS mouse model was created in two different strains of mice. ICR female mice were used to assess the changes in the estrous cycle, body weight, and pregnancy rate, whereas, GnRH-GFP tagged mice were used for electrophysiological recording. Letrozole 30 µg/ml stock solution was dissolved in 4% aqueous solution of carboxymethylcellulose (CMC) and stored at 4°C. As shown in **Figure 1A**, ICR female mice of postnatal day (PND) 21 were divided into two groups. For the control group, mice were orally fed with CMC alone by gavage and for the drug-treated group, 1 µg/g body weight of letrozole was orally fed by gavage once a day for 21 days to induce polycystic ovary (8, 16). Body weight was measured every week during the feeding period. For electrophysiological recording, five batch of GnRH-GFP-tagged female mice of PND 21 were orally fed by gavage with 1 µg/g of letrozole dissolved in CMC (n = 6 in each batch) or CMC alone (n = 5 in each batch) for 21 days as shown in **Figure 2A**. Then, one mouse from either control or letrozole-fed group was alternatively decapitated on daily basis between 11:00 AM to 12:00 PM UTC + 9:00 (Universal Time Coordinate) for the electrophysiological assessment.

### Estrous cycle and fertility assessment

The estrous cycle of the mice was assessed for two weeks after completion of letrozole feeding. Estrous phases were determined by monitoring the morphology of the vaginal smear under light microscopy as described by previous study (32). Briefly, vagina was gently flushed 2-3 times with Phosphate Buffered Saline (PBS), and the final flush was collected in the pipette tip and then smeared on the clean glass slides. The dried samples were stained using (0.2%) methylene blue and examined under a light microscope. The phases of the estrus cycle were then identified by cytological features. The predominant presence of nucleated epithelial in individuals or clusters confirmed the proestrus phase. In addition, the estrous phase showed the predominance of cornified epithelial cells, whereas the metaestrous phase showed an increase in the number of leukocytes with epithelial cells. Similarly, the diestrous phase had leukocytes as predominant cells. Metaestrous and diestrous stages showed identical characteristics; thus, the data were pooled as simply diestrous.

For fertility assessment, female mice (PND 60) from the control group (n = 10) and letrozole-fed group (n = 5) were paired with fertile males (a total of 15 breeding pairs), and copulatory plugs were checked daily. Within 4 to 5 days, all mice had a copulatory plug, which was marked as gestational day 1, and all the males were removed from the cages.

### Preparation of brain slices and electrophysiology

Coronal brain slices were prepared using the same procedure as described in a previous study (33). GnRH-GFP tagged female mice from control and letrozole-fed groups were decapitated between 11:00 AM to 12:00 PM UTC + 9:00 (Universal Time Coordinate). Their brains were removed quickly and placed in an ice-cold artificial cerebrospinal fluid (ACSF) with the following composition (in mM): 126 NaCl, 2.5 KCl, 2.4 CaCl_2_, 1.2 MgCl_2_, 11 D-glucose, 1.4 NaH_2_PO_4_, 25 NaHCO_3_ and pH maintained to 7.3 - 7.4 by bubbling with 95% O_2_ and 5% CO_2_. Coronal slices (180 µm thickness) containing the preoptic hypothalamic area were cut using a vibratome (VT1200S; Leica biosystem, Wetzlar, Germany) in the ice-cold ACSF. Slices were then allowed to recover in oxygenated ACSF before being transferred to the recording chamber. The images of coronal slices were viewed under an inverted microscope (BX51WI; Olympus, Tokyo, Japan) and displayed on a video monitor. GnRH neurons were identified using 40X objective lens by brief fluorescence illumination and patched under Nomarski differential interference contrast optics. Patch pipettes were pulled from thin-wall borosilicate glass-capillary tubing (PG52151-4; WPI, Sarasota Bay, FL, USA) on a Flaming/Brown puller (P-97; Sutter Instruments Co., Novato, CA, USA). The patch pipette was back-filled with an internal solution containing (in mM): 140 KCl, 1 CaCl_2_, 1 MgCl_2_, 10 HEPES, 4 MgATP, and 10 EGTA (pH 7.3 with KOH). The tip resistance of the patch pipette filled with an internal solution was approximately 4 to 6 MΩ. The electrode potential was nullified before the gigaseal formation. Then the neurons were voltage clamped at -60 mV, and a gentle suction was applied to rupture the cell’s membrane to make a whole-cell configuration. The whole-cell patch-clamp recordings were performed under voltage clamp mode using the Axopatch 200B amplifier (Molecular Devices, San Jose, CA, USA). The changes in membrane current were sampled online using a Digidata 1322A interface (Molecular Devices) connected to a desktop PC.

For perforated patch-clamp recordings, gramicidin (Sigma-Aldrich, St. Louis, MO, USA) was first dissolved in dimethyl sulfoxide (DMSO; Sigma-Aldrich) to a concentration of 2.5–5 mg/ml. Then diluted to a final concentration of 2.5–5 μg/ml in the pipette solution and sonicated for 20 min before the use. Access resistance was monitored in the initial experiments, and experiments began when the resistance stabilized at 50–90 mΩ. Typically, after 15–30 min of gigaseal formation, the cell’s resting membrane potential (RMP) reaches a stable level below −45 mV. Any spontaneous membrane rupture was evident by sudden increases in membrane potentials above 0 mV. All the recordings were made at room temperature.

### Chemicals

All the chemical reagents for ACSF and pipette solutions, including letrozole (L6545), γ-aminobutyric acid (GABA) (A2129), 4,5,6,7-tetrahydroisoxazolo[5,4-c]pyridine-3-ol (THIP) hydrochloride (T101), tetrahydrodeoxycorticosterone (THDOC) (P2016), bicuculline (14340), kainic acid (K0250), dehydroepiandrosterone (DHEA) (252805) and metformin (PHR1084) were purchased from Sigma-Aldrich (St. Louis, MO, USA) whereas, muscimol (0289) was obtained from Tocris Bioscience. In addition, 2,4-Thiazolidinedione (SC-216281) was purchased from Santa Cruz Biotechnology (Finnell Street, Dallas). Kisspeptin-10 (048-56) was obtained from Phoenix Pharmaceuticals, INC (Burlingame, CA, USA). All the chemicals were dissolved in distilled water except THDOC, Thiazolidinedione, and DHEA, which were dissolved in DMSO. Stocks were diluted (usually 1,000-fold) in ACSF to desired final concentrations before bath application.

### Data and statistical analysis

All statistical values were expressed as mean ± standard error of the mean. The Shapiro-Wilk test was used to determine whether the data had a normal distribution. The unpaired *t*-test was used to compare the data from a normal distribution, while the Mann-Whitney test was used to compare data from a non-normal distribution. In addition, the percentage of the estrous cycle and pregnancy outcomes were analyzed using the chi-square test. Statistical analysis was performed in Origin software (OriginLab Corp, Northampton, MA, USA), and the tests performed are indicated in the figure legends. Statistical significance was defined as a *p*-value < 0.05. Acquisition and subsequent analysis of the acquired data were performed using Clampex software (Axon Instruments). The traces were plotted using Origin 8 software (OriginLab Corp., Northampton, MA, USA).

## Results

### Letrozole feeding disrupts the estrous cyclicity and impairs fertility

In this study, all control females had 4 to 5 days of the normal estrous cycle, but letrozole-fed mice showed irregularity in the estrous cycle (**Figures 1B, C**). The pie chart shows the average percentage of three phases of the estrous cycle in control and letrozole-fed mice in two weeks period (**Figure 1D**). In both groups, the proestrous phase remained almost constant, but the letrozole-fed mice had a shorter estrous phase (control: 28.6%; letrozole: 12.8%) and prolonged diestrous phase (control: 44.3%; letrozole: 60.0%) than control. Next, the average days spent in three phases of an estrous cycle that occurred in two weeks were compared. Control and letrozole-fed mice had nearly similar proestrus duration (control: 1.28 ± 0.12 days, letrozole: 1.46 ± 0.14 days, p > 0.05, unpaired *t*-test) whereas the estrous phase duration was reduced (control: 1.28 ± 0.12 days, letrozole: 0.75 ± 0.17 days, *p < 0.05, unpaired *t*-test), and the diestrous phase duration was prolonged (control: 2.06 ± 0.22 days, letrozole: 3.23 ± 0.45 days; *p < 0.05, unpaired *t*-test) in the letrozole-fed mice (**Figure 1E**). Additionally, the body weight of letrozole-fed mice was significantly increased in the third week of letrozole treatment (control: 24.6 ± 0.29 gm, n = 15; letrozole: 26.3 ± 0.27 gm, n = 15; *p < 0.05, unpaired *t*-test, **Figure 1F**).

PCOS is considered to have a significant impact on pregnancy outcomes. Compared with control females, letrozole-fed mice had a lower pregnancy rate. On gestational day 21, 9 out of 10 (90%) control females delivered pups, while 2 out of 5 (40%) letrozole-fed females delivered pups (p < 0.05, chi-square test, **Figure 1G**). Furthermore, letrozole-fed females delivered less number of pups per litter (n = 7.0 ± 2.0, N = 2) compared to controls (n = 9.4 ± 0.76, N = 9) but this difference was statistically insignificant (figure not shown, ‘n’ represents pups number and ‘N’ represents litter number).

### Letrozole feeding suppresses GABA and glutamate-mediated responses on GnRH neurons

GABA and glutamate receptor-mediated responses were recorded using the whole-cell patch-clamp technique from GnRH neurons distributed in the hypothalamic preoptic regions of GnRH-GFP- mice. To see if there were any variations in the response produced by excitatory neurotransmitters in letrozole-fed mice, we compared the responses elicited by GABA (100 µM), muscimol (3 µM), and kainate (10 µM) in control and letrozole-fed groups. **Figure 2A**, shows the experimental paradigm for the electrophysiological assessment. The traces of GABA-induced inward current from control and letrozole-fed mice are shown in **Figure 2B**. With respect to control, mean inward current induced by GABA was remarkably decreased in letrozole-fed mice (control: -970.0 ± 164.0 pA, n = 15; letrozole: -251.0 ± 46.0 pA, n = 20; *p < 0.05, unpaired *t*-test, **Figure 2C**). Furthermore, GABA_A_ receptor agonist muscimol-mediated response was dramatically reduced in the letrozole-fed mice (**Figure 2D**). The mean inward current induced by muscimol was significantly lower in letrozole-fed mice compared to the control (control: -453. ± 56.0 pA, n = 12; letrozole: -276.0 ± 28.19 pA, n = 18; *p < 0.05, unpaired t-test, **Figure 2E**). Furthermore, the glutamate agonist kainate had a negligible effect on the GnRH neurons of letrozole-fed mice (**Figure 2F**). The mean inward current induced by kainate was significantly decreased in letrozole-fed mice compared to control (control: -18.3 ± 2.94 pA, n = 13; letrozole: -8.22 ± 1.73 pA, n = 19, ^#^p < 0.05, Mann-Whitney test, **Figure 2G**).

### Letrozole feeding reduces neurosteroid-mediated actions on GnRH neurons

**Figure 3:**
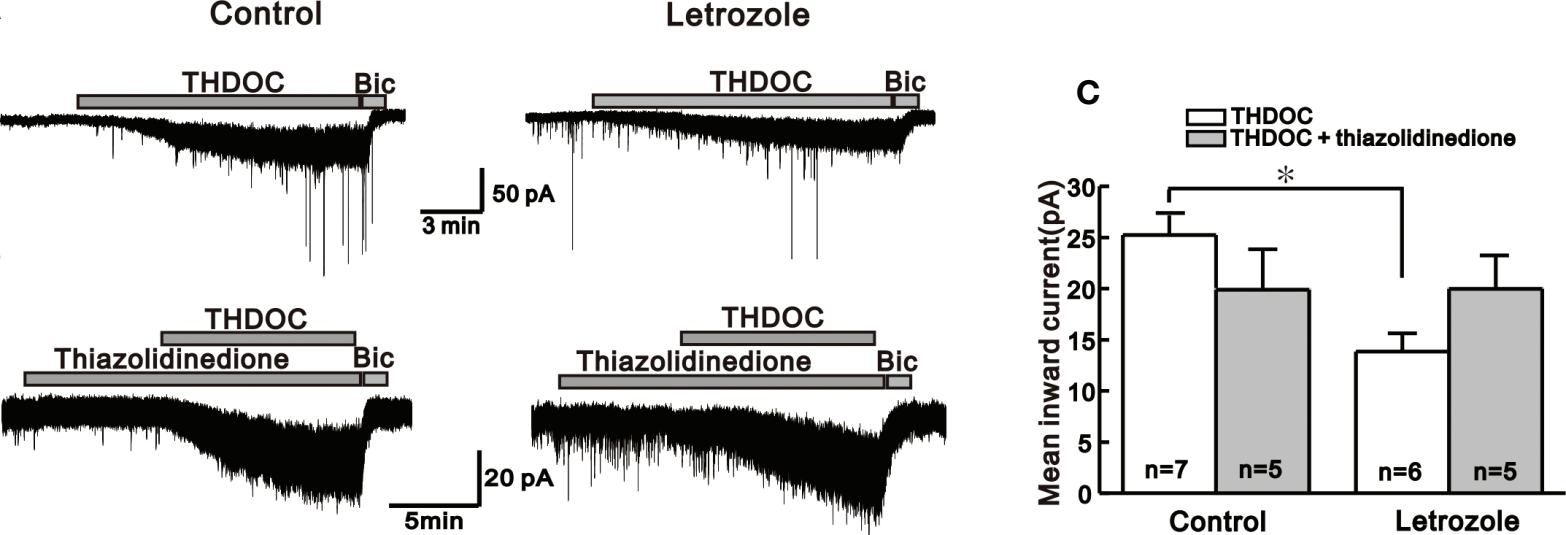
Neurosteroid THDOC induced response in the control and letrozole-fed mice. **(A)** Representative traces for the neurosteroid THDOC-mediated responses on GnRH neurons from the control and letrozole-fed mice. **(B)** Representative traces for neurosteroid THDOC-mediated responses on GnRH neurons of control and letrozole-fed mice in the presence of thiazolidinedione, a medicine used to treat type 2 diabetes and PCOS. **(C)** Histogram compares the mean inward currents induced by THDOC and THDOC with thiazolidinedione on GnRH neurons from the control and letrozole-fed mice, respectively. (*p < 0.05, unpaired *t*-test).

Next, we compared the response induced by bath application of endogenous neurosteroids on GnRH neurons among control and letrozole-fed mice. In GnRH neurons, neurosteroids activates the extrasynaptic GABA_A_ receptors and mediates tonic form of GABAergic signaling (34). Under whole-cell voltage-clamp mode, neurosteroids THDOC and THIP mediated response on GnRH neurons were recorded from control and letrozole-fed mice. THDOC, a potent allosteric modulator of GABA_A_ receptor, elicited a larger response in GnRH neurons from the control (-25.2 ± 1.90 pA, n = 7) compared to the letrozole-fed mice (-13.8 ± 1.79 pA, n = 6, *p < 0.05, unpaired *t*-test, **Figures 3A, C**). Next, the effect of thiazolidinedione on THDOC-induced response in GnRH neurons of the control and letrozole-fed mice was assessed (**Figure 3B**). Thiazolidinedione is a medicine used for treating type 2 diabetes and PCOS (35). Pretreatment of the slice with thiazolidinedione for 10 to 15 minutes resulted in slight decrease of THDOC-mediated response in GnRH neurons from the control group (THDOC: -25.2 ± 1.90 pA, n = 7, THDOC + Thiazolidinedione: -19.9 ± 3.95 pA, n = 5; p > 0.05, unpaired t-test, **Figure 3C**). However, in a letrozole-fed mice, thiazolidinedione pretreatment did not change the THDOC-mediated response in GnRH neurons (THDOC: -13.8 ± 1.79 pA, n = 6, THDOC + thiazolidinedione: -19.9 ± 3.33 pA, n = 5; p > 0.05, unpaired t-test, **Figure 3C**).

**Figure 4:**
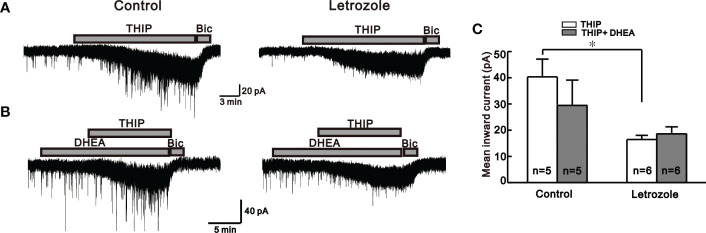
Neurosteroid THIP-induced responses in the control and letrozole-fed mice. **(A)** Representative traces for neurosteroid THIP mediated responses on GnRH neurons from control and letrozole-fed mice. **(B)** Representative traces for neurosteroid THIP-mediated responses on GnRH neurons from control and letrozole-fed mice in the presence of DHEA, a PCOS inducer. **(C)** Histogram comparing the mean inward currents induced by THIP and THIP with DHEA on GnRH neurons from control and letrozole-fed mice, respectively. (*p < 0.05, unpaired *t*-test).

Similarly, another neuroactive steroid, THIP, which activates extrasynaptic GABA_A_ receptors containing δ-subunit, exerts a similar response as THDOC in control and letrozole-fed mice. Bath application of THIP-induced larger response in the GnRH neurons of control (-40.4 ± 6.74 pA, n = 5) compared to the letrozole-fed mice (-16.4 ± 1.59 pA, n = 6, *p < 0.05, unpaired *t*-test, **Figure 4A, C**). Next, the effect of DHEA, a PCOS inducer (36), on THIP-induced response in GnRH neurons of the control and letrozole-fed mice was assessed (**Figure 4B**). Pre-treatment of the slice with DHEA for 10 to 15 minutes resulted in the decrease of THIP- mediated response in GnRH neurons of control mice (THIP: -40.4 ± 6.74 pA, n = 5, THIP + DHEA: -29.2 ± 3.44 pA, n = 5; p > 0.05, unpaired t-test, **Figure 4C**). However, pretreatment with DHEA did not affect the THIP response in the letrozole-fed mice (THIP: -16.4 ± 1.59 pA, n = 6, THIP + DHEA: -18.4 ± 1.70 pA, n = 6; p > 0.05, unpaired *t*-test, **Figure 4C**).

### Letrozole feeding decreases the kisspeptin sensitivity on GnRH neurons

**Figure 5:**
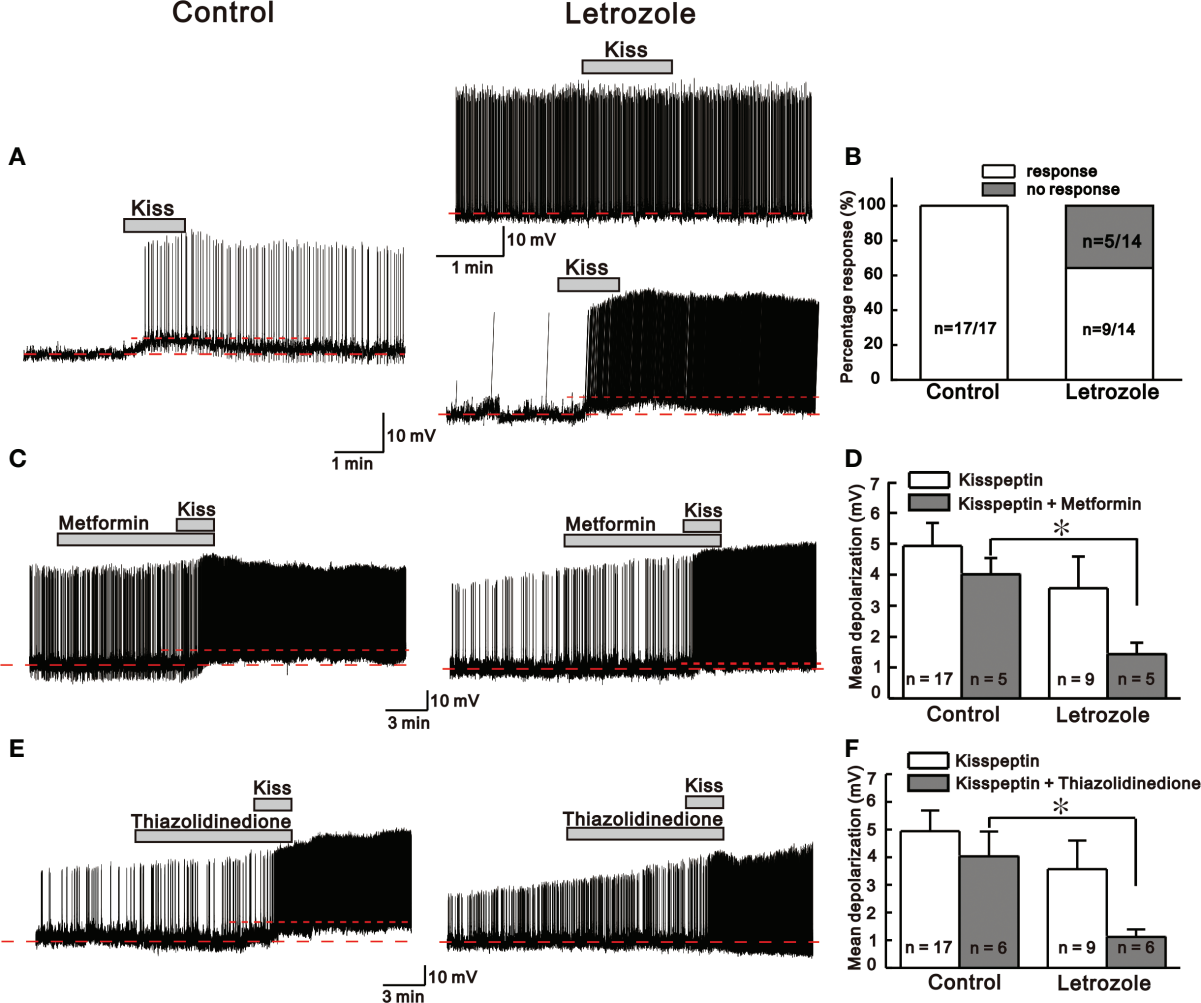
Kisspeptin (KP)-mediated responses between control and letrozole-fed mice. **(A)** Representative traces for KP-mediated responses on GnRH neurons from control and letrozole-fed mice. **(B)** A histogram showing the percentage of GnRH neurons responding to KP in the control and letrozole-fed mice. **(C, E)**. Representative traces for KP-induced responses on GnRH neurons from control and letrozole-fed mice in the presence of metformin and thiazolidinedione (both used as a medicine to treat type 2 diabetes and PCOS), respectively. **(D, F)**. Histogram comparing the mean depolarization induced by KP in the presence of metformin and thiazolidinedione on GnRH neurons from control and letrozole-fed mice, respectively. (*p < 0.05, unpaired *t*-test).

Next, to investigate whether letrozole affects the kisspeptin sensitivity on adult GnRH neurons, 30 nM kisspeptin was bath applied in perforated patch-clamp mode. Here, we compared the kisspeptin sensitivity on adult GnRH neurons from control and letrozole-fed mice. In control mice, all tested GnRH neurons (n = 17, 100%) responded to kisspeptin with either membrane depolarization or membrane depolarization accompanied by the continuous firing of action potential (AP). However, only 64.3% (n = 9/14) of GnRH neurons from letrozole-fed mice responded to kisspeptin with similar characteristics to control, while the remaining neurons did not respond to kisspeptin. A significant difference was found in the percentage of GnRH neurons showing excitation between the control and letrozole group (p < 0.05; chi-square test, **Figure 5B**). **Figure 5A** represent the traces for kisspeptin-induced excitation of GnRH neurons from control and letrozole-fed mice. Although the percentage of GnRH neurons responding to kisspeptin was lower in letrozole-fed mice, the mean membrane depolarization induced by kisspeptin was not significantly different from control (control: 4.94 ± 0.74 mV, n = 17; letrozole: 3.57 ± 1.03 mV, n = 9; p > 0.05, unpaired *t*-test).

Next, kisspeptin evoked excitation on GnRH neurons from both the control and letrozole-fed mice was recorded in the presence of metformin and thiazolidinedione, both drugs are used in treatment of PCOS and type 2 diabetes mellitus (35). Metformin and thiazolidinedione were bath applied to the GnRH neurons for at least 10 to 15 min before the kisspeptin perfusion. In the presence of metformin and thiazolidinedione, all the GnRH neurons from control mice responded to kisspeptin with membrane depolarization accompanied by spontaneous AP firings, whereas, in letrozole-fed mice, the majority of the neurons displayed AP firing alone (**Figures 5C, E**). In the presence of metformin, kisspeptin evoked depolarization was significantly smaller in the letrozole-fed mice compared to control (control: 4.02 ± 0.5 mV, n = 5, letrozole: 1.43 ± 0.37 mV, n = 5; *p < 0.05, unpaired *t*-test, **Figure 5D**). Similarly, in the presence of thiazolidinedione, kisspeptin evoked membrane depolarization was also significantly smaller in the letrozole-fed mice compared to the control (control: 4.02 ± 0.89 mV, n = 6, letrozole: 1.12 ± 0.26 mV, n = 6; *p < 0.05, unpaired *t*-test, **Figure 5F**).

## Discussion

In this study, letrozole feeding resulted in disrupted estrous cycles, reduced fertility, and increased body weight in female mice. Letrozole-fed mice had irregular estrous cycles, with a highly disrupted estrous phase. In addition, letrozole-fed mice demonstrated reduced pregnancy outcomes even though a copulatory plug was detected in females from both groups of breeding pairs. Previous findings show impaired fertility in the letrozole-induced PCOS models (10), as well as abnormalities in the ovarian and uterine tissues (15, 37) and the development of cystic ovary (15, 38). These reported findings could support the possibility of impaired fertility even though a copulatory plug was detected in our letrozole-fed mice.

Validity of the letrozole-induced PCOS model in rodents has been well-studied in previous research studies with a possibility of restoration of normal estrous cycling and ovulation at an average time frame of nearly 4-6 weeks after letrozole-fed stop day (10, 14, 15, 39, 40). Here, the electrophysiological data collection, estrous cycle, and fertility assessment were done within four weeks of letrozole-fed stop day, suggesting that the results observed in the study were due to letrozole treatment.

Letrozole-induced PCOS mouse model in GnRH-GFP tagged mice allowed us to explore the various neurotransmitter system of GnRH neurons crucial for normal functioning of reproduction and fertility. Our electrophysiological findings revealed the reduced response of neurotransmitters like GABA, glutamate and kisspeptin in letrozole-fed mice. Similarly, neurosteroids THDOC and THIP, the potent modulators of extrasynaptic GABA_A_ receptors, had a diminished effect on GnRH neurons of letrozole-fed mice.

Most PCOS animal models showed increased GnRH pulse and LH pulse frequency (7); however, some reported suppressed and no effect on GnRH pulse (41, 42). For example, prenatally androgenized (PNA) PCOS and letrozole-fed rodents revealed increased LH pulse frequencies (10, 30, 43, 44). In addition, letrozole-induced PCOS had significantly elevated serum testosterone level and LH (10). Contrary to these findings, letrozole suppressed the GnRH pulses and the mean release of GnRH in rhesus monkeys (41). In addition, chronic DHT exposure had no effect on LH pulse frequency or GABAergic inputs to GnRH neurons (42). It is reported that the timing of preovulatory LH surge is probably controlled by GABA receptors (45, 46). Typically GABA_A_ acts as an inhibitory neurotransmitter; however, its role is debatable in the case of GnRH neurons (47, 48). In mature GnRH neurons, the excitatory effect of GABA_A_ receptors is due to elevated intracellular chloride levels (49).

In the PNA PCOS mouse model, higher GABAergic effect on GnRH neurons has been reported (28). However, in our experimental conditions, we report lower GABA_A_ receptor activity on GnRH neurons in a PCOS mouse model induced by letrozole. GABA_A_ agonist muscimol-mediated effects on GnRH neurons were significantly reduced in our letrozole-induced PCOS mice compared to the control group. Furthermore, the neurosteroids THDOC and THIP, which exert their rapid action on GnRH neurons through allosteric modulation of GABA_A_ receptors (50), had a lower influence on GnRH neurons of letrozole-fed mice. Similar to our findings, *Porter et al.* discovered reduced GABAergic inputs to preoptic GnRH neurons in a prenatal testosterone PCOS model (51). Similarly, letrozole and testosterone-induced PCOS models showed reduced GABA levels in the hypothalamus (29, 30). Clinical observation also reports lower serum GABA levels in Egyptian infertile females with PCOS (52). With these observations, it could make sense that letrozole-fed mice had decreased GABA_A_ receptor-mediated activity on GnRH neurons. Furthermore, we also observed that type 2 diabetes medicine thiazolidinedione partially recovered suppressed GABA_A_ receptor activity in GnRH neurons of the letrozole-fed mice. In contrast, DHEA, a PCOS inducer, reduced GABA_A_ receptor activity slightly in control groups but not in letrozole-fed mice. These findings show that letrozole-induced PCOS significantly alters not only the phasic GABA_A_ receptor signaling activated by muscimol but also the extrasynaptic GABA_A_ receptor-mediated actions on GnRH neurons.

GnRH neurons express functional receptor subunits for ionotropic glutamate receptors (22) and kisspeptin receptors (53). Stimulating these receptors excites the GnRH neurons (54, 55). Letrozole-fed mice also displayed diminished ionotropic glutamate and kisspeptin receptor activation. The response to the ionotropic glutamate receptor agonist kainate was significantly lower in the GnRH neurons from letrozole-fed mice than control mice. GnRH neurons express the Kiss1/GPR-54 receptor which has high affinity for kisspeptin (53, 56). Kisspeptin is a potent GnRH neuron activator and induces intense and prolonged excitation with action potential firing in 100% adult GnRH neurons (54). Kisspeptin excited all tested neurons in control mice but only 64% of GnRH neurons from letrozole-fed mice. However, these two groups showed comparable membrane depolarization on kisspeptin exposure. This finding suggests that letrozole feeding can decrease the sensitivity of GnRH neurons to kisspeptin.

Kisspeptin signaling plays a critical role in the physiological regulation of the HPG axis *via* the KISS1R, but its role in the pathogenesis of PCOS is unknown. Most clinical observations suggest higher serum kisspeptin levels in PCOS patients, with few showing no significant difference from non-PCOS counterparts (57–59). Several studies show contradictory findings for KISS1R expression between PCOS animals and control. For example, prenatal DHT-induced PCOS and estradiol-induced PCOS showed increased KISS1 expression (60). Kisspeptin-positive cells were highly expressed in the arcuate nucleus (ARC) in the letrozole-induced PCOS model but decreased in the anteroventral periventricular nucleus (AVPV) (61). Similarly, another study reported higher KISS1 mRNA expression across ARC instead of the AVPV (62). However, some findings suggest decreased KISS1R mRNA and kisspeptin immunoreactivity in the hypothalamus of a DHT-induced PCOS model (63). Furthermore, KISS1 gene was reduced in the hypothalamus of testosterone-induced PCOS (11). These findings mentioned above raise the possibility that kisspeptin functions differentially in different PCOS models. Our electrophysiological data showed reduced kisspeptin sensitivity on GnRH neurons in letrozole-fed mice. In addition, anti-hyperglycaemic drugs metformin and thiazolidinedione significantly affected the kisspeptin-induced membrane depolarization on GnRH neurons of letrozole-fed mice. This is the first electrophysiological data to record kisspeptin sensitivity on GnRH neurons in a letrozole-induced PCOS mouse model. Further studies on other PCOS models are required to ascertain the role of kisspeptin signaling in GnRH neurons in PCOS conditions.

The etiology of PCOS’s pathophysiology is largely unknown. For the analytical exploration of PCOS, research on various animal PCOS models at different levels of HPG axis has been carried. Study at the hypothalamic level could facilitate to understand pathophysiology and therapeutic targets to PCOS. PCOS has been linked to the alterations in the neurotransmitters and neuropeptides that regulate the GnRH neurons and GnRH/LH pulsatility (29, 30). An imbalance in the neurotransmitter system can impact the neuroendocrine axis regulating reproduction. Suppression of GABA, glutamate, and kisspeptin transmission in GnRH neurons, as well as irregular estrous cycle, and fecundity in letrozole-fed mice, indicates a disruption of the neuroendocrine axis regulating reproductive physiology. However, we did not undertake hormone assays for LH, FSH, estradiol, progesterone, and testosterone in the letrozole-fed and control groups.

To summarize, we found that the neurotransmission mediated by GABA_A_, kainate, and kisspeptin in the GnRH neurons was suppressed when the mice were orally fed with letrozole, a non-steroidal aromatase enzyme inhibitor. Furthermore, mice also displayed reproductive abnormalities similar to other PCOS models. This indicates the involvement of GnRH-regulatory neurotransmitters in the pathogenesis of PCOS induced by letrozole. The electrophysiological data are limited and warrant further study in different PCOS models to know the etiology of neuroendocrine disruption in PCOS.

## Data availability statement

The raw data supporting the conclusions of this article will be made available by the authors, without undue reservation.

## Ethics statement

The animal study was reviewed and approved by Institutional Animal Care and Use Committee of Jeonbuk National University, CBNU 2016-64, and CBNU 2020-0122.

## Author contributions

PB and SR performed the experiments, analyzed the data, and wrote the draft. JB contributed to reviewing and editing the draft. DC and SH conceptualized and design the study and completed the manuscript. All authors contributed to the article and approved the submitted version.
